# Uterine Anaphylaxis Due to Subcutaneous Immunotherapy (SCIT) for Inhalant Allergens: A Case Series

**DOI:** 10.7759/cureus.42526

**Published:** 2023-07-27

**Authors:** Malika P Ganguli, Shayan Sinha, Vesselin Dimov, Frank Eidelman

**Affiliations:** 1 Medicine, Ross University School of Medicine, Bridgetown, BRB; 2 Internal Medicine, Cleveland Clinic Florida, Weston, USA; 3 Allergy and Immunology, Cleveland Clinic Florida, Weston, USA

**Keywords:** systemic reaction, uterine cramping, seasonal allergies, allergen, inhalant allergies, allergy shots, subcutaneous immunotherapy (scit), allergy, anaphylaxis, uterine

## Abstract

Subcutaneous immunotherapy (SCIT), commonly known as “allergy shots,” aims to achieve a clinical tolerance for allergens that cause symptoms of allergic rhinoconjunctivitis, allergic asthma, or insect sting allergies. Systemic reactions to SCIT are classified in severity from one organ system (grade 1) to anaphylaxis that potentially may have a fatal outcome (grade 5). Uterine cramps fall under grade 2, and they are rarely reported during SCIT. In this study, we report four cases of uterine anaphylaxis following SCIT for environmental allergens with symptoms such as severe lower abdominal cramping resembling menstrual cramps. Patients also experienced urticaria, angioedema, diffuse erythema, and flushing. None of the patients experienced uterine bleeding. To the best of our knowledge, this is the largest case series reporting this reaction to SCIT. We accompany this finding with a review of the literature on this rare but interesting topic.

## Introduction

Subcutaneous immunotherapy (SCIT), also known as allergy shots, is a well-known procedure done in clinical practice by an allergist where extracts of an allergen are injected into a patient to slowly reduce and eliminate the resultant hypersensitivity reactions [1]. Side effects of SCIT can be local or systemic reactions. Systemic reactions to SCIT are classified into five grades, occurring in up to 0.6% of patients [2]. A reaction to a single organ system is considered grade 1. Grade 2 and 3 reactions involve more than one organ system or the presence of asthma, cardiovascular symptoms, or gastrointestinal (GI) symptoms [3]. Severe reactions and hemodynamic instability is classified as grade 4. Grade 5 is death. The grading system is further subclassified with letters A, B, C, D, and Z. Letter A is a reaction lasting less than five minutes, letter B for 5-10 minutes, letter C for 10-20 minutes, letter D for greater than 20 minutes, and letter Z if epinephrine was not administered [3].

Uterine cramps are classified under grade 2; however, some literature recommends upgrading uterine cramping to grade 3 [1,3]. As a rare side effect of SCIT, it is poorly reported in the medical literature. This report is an important addition to the medical literature. As to the best of our knowledge, this is the largest case series reporting this finding in patients undergoing SCIT for inhalant allergens.

This article was previously posted as an abstract in the Journal of Allergy and Clinical Immunology in February 2016.

## Case presentation

In this report, we describe four cases of uterine anaphylaxis 30-45 minutes after SCIT identified between 2010 and 2014. None of the patients had a new allergen added to therapy, there were no changes in suppliers for allergen extracts, and all allergen extracts were standardized. None had a history of dysmenorrhea nor bleeding per vagina during anaphylaxis events. SCIT allergens with doses and concentrations are listed in Table 1.

**Table 1:** SCIT allergens with concentrations and doses SCIT: subcutaneous immunotherapy

	Anaphylactic event #1	Anaphylactic event #2	Anaphylactic event #3
Patient 1	No formulation data available. The patient experienced diffuse pruritus with urticaria and severe (9/10) abdominal pain	-	-
Patient 2	Dust mite 0.150 mL (1:1), dog 0.150 mL (1:1), trees 0.150 mL (1:1), grasses 0.150 mL (1:1). The patient experienced nausea, diffuse urticaria, eyelid angioedema, and severe abdominal pain.	Cat 0.500 mL (1:1), trees 0.500 mL (1:1), weeds 0.400 mL (1:1), grasses 0.150 mL (1:1). The patient experienced facial flushing and urticaria with no abdominal cramping.	-
Patient 3	Dust mite 0.300 mL (1:1), cockroach 0.300 mL (1:1), feathers 0.300 mL (1:1), trees 0.300 mL (1:1), weeds 0.300 mL (1:1), grasses 0.300 mL (1:1). The patient experienced urticaria and uterine cramping.	Dust mite 0.150 mL (1:1), cockroach 0.150 mL (1:1), feathers 0.150 mL (1:1), trees 0.150 mL (1:1), weeds 0.150 mL (1:1), grasses 0.150 mL (1:1). The patient experienced diffuse urticaria, redness, and uterine cramping.	-
Patient 4	Dust mite 0.500 mL (1:1). The patient experienced uterine cramping.	Dust mite 0.500 mL (1:1). The patient experienced facial swelling and uterine cramping.	Dust mite 0.300 mL (1:1). The patient experienced sole uterine cramping.

Patient 1 is a 43-year-old female with a past medical history of allergic rhinitis treated with 10 mg cetirizine per day and azelastine nasal spray twice per day. At the time of anaphylaxis, she was in the buildup phase of SCIT (exact concentrations of immunotherapy were not available). She was pretreated with levocetirizine and montelukast. However, 30 minutes after injection, she reported diffuse pruritus and severe abdominal pain of 9/10 severity. On physical examination, hives were noted on the anterior chest with tenderness on abdominal palpation in the right lower quadrant, left lower quadrant, and suprapubic areas. Vital signs were within normal limits. The patient received intramuscular epinephrine, diphenhydramine, and ranitidine and reported rapid improvement. She opted to report to the emergency department for further testing. Chest X-ray showed no acute infiltrates or pulmonary embolisms. Computed tomography (CT) with and without contrast of the abdomen and pelvis did not report any renal stones, bowel obstructions, or pelvic abnormalities.

**Figure 1:**
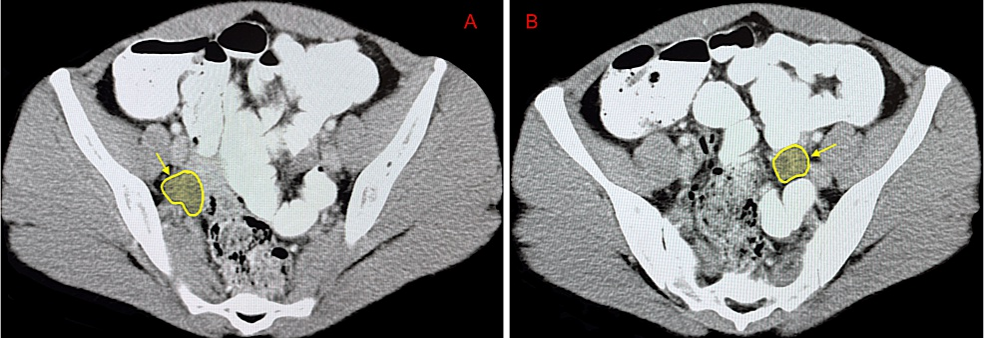
CT of the abdomen and pelvis with contrast revealing bilateral ovarian cysts (yellow arrow) in patient 2 A: The right ovarian cyst, measuring 2.4 cm. B: The left ovarian cyst, measuring 1.6 cm. CT: computed tomography

Patient 2 is a 39-year-old female with a past medical history of allergic rhinitis treated with 180 mg fexofenadine. The patient had two anaphylactic reactions to SCIT (Table 1). In her first reaction, she reported nausea, diaphoresis, diffuse hives located on her entire body, angioedema of the eyelids, and severe lower abdominal pain resembling menstrual cramps 25 minutes after the SCIT injection. Vital signs were within normal limits. She received intramuscular epinephrine and diphenhydramine with relief of symptoms within 20 minutes, and SCIT dosing was reduced to 0.3 mL (1:1) for all allergens with a plan to rebuild dosing monthly. Two months later, the patient had a second systemic reaction with facial flushing, hives on the back of the neck, and no abdominal cramping. These symptoms occurred 30 minutes after injection (Table 1). She was treated with intramuscular epinephrine and diphenhydramine and improved within minutes. She was advised to premedicate with fexofenadine one hour prior to her next SCIT series, which would be reduced to a dose of 0.2 (1:1) with split doses. It should be noted that this patient had a known gynecological history of cervical dysplasia treated with a loop electrosurgical procedure in 1997 and ovarian cysts. Her ovarian cysts were evaluated by CT of the abdomen and pelvis (Figure 1) suggesting physiological bilateral ovarian cysts, likely follicular, ranging from 1.6 to 2.4 cm, and may be hemorrhagic. It is unlikely that her gynecological history was related to her multisystem SCIT reactions.

Patient 3 is a 34-year-old female with a history of asthma and allergic rhinitis treated with a 90 mcg albuterol inhaler, 50 mcg fluticasone nasal spray, 180 mg fexofenadine per day, and 10 mg montelukast per day. She had two anaphylactic reactions to SCIT (Table 1). In her first reaction, she experienced uterine cramping and urticaria of the neck. Vital signs were within normal limits. This reaction took place during the peak of tree and grass pollen season, possibly contributing to the onset and severity of the reaction. She was treated with intramuscular epinephrine, diphenhydramine, and ranitidine with resolution of symptoms within an hour. Her dose was subsequently reduced from 0.300 (1:1) to 0.200 (1:1). One year later, she experienced a systemic reaction with diffuse urticaria, redness, and lower abdominal cramping resembling a severe menstrual cramp. On physical examination, she was diaphoretic and cool with a pulse of 58 beats per minute. She received intramuscular epinephrine, diphenhydramine, ranitidine, and albuterol nebulizer and was placed in a supine position for observation. She improved within a period of one hour and was recommended to premedicate with cetirizine and montelukast one hour prior to her future SCIT injections. In reviewing this patient’s SCIT history, it was noted that she missed several doses of immunotherapy, perhaps contributing to her several anaphylactic events. She later discontinued SCIT due to the frequency of anaphylaxis.

Patient 4 is a 40-year-old female with a medical history of allergic rhinitis treated with 10 mg montelukast per day and a 137-50 mcg azelastine-fluticasone combination nasal spray. In prior SCIT regimens, this patient was recorded to have multiple systemic reactions without uterine cramping, experiencing dry cough with itching of the throat. She first experienced lower abdominal cramps when administered SCIT at 0.500 (1:1) to dust mite allergens (Table 1), at which time she was treated with intramuscular epinephrine and diphenhydramine. SCIT was reduced to 0.250 (1:1) and tolerated well. A dose titration to 0.5 (1:1) invoked another allergic reaction, reporting facial swelling and uterine cramping. She was treated again with intramuscular epinephrine and diphenhydramine. The dose was reduced again to 0.200 (1:1) and tolerated well. An attempt to increase dosages again to 0.300 (1:1) caused another reaction with sole uterine cramping. She was vitally stable during all of her anaphylactic events and premedicated with cetirizine and montelukast. She was noted to miss one dose of immunotherapy during her treatment course. She ultimately decided to terminate SCIT due to concerns regarding pregnancy with SCIT-induced uterine cramping.

## Discussion

Uterine cramping is a known side effect of SCIT, likely underreported in the literature. It is perhaps under-acknowledged by healthcare providers as a component of biphasic anaphylactic reactions when patients are not undergoing SCIT [4,5]. It has also been reported as a potential symptom of anaphylaxis in pregnancy, in addition to other rare presentations of allergy such as vulvar and vaginal itching and vaginal bleeding [5]. It is also possible that uterine cramping is not recognized as a symptom if the patient reported generalized abdominal cramping, which may be secondary to gastrointestinal symptomatology seen in anaphylaxis [3]. The patients in this case report did not report any gastrointestinal symptoms such as nausea, vomiting, or diarrhea. Furthermore, they specified that their symptoms were in the lower abdomen and pelvis mimicking a severe menstrual cramp.

A review of the literature revealed very few case reports of uterine anaphylaxis with SCIT. One abstract reported sole severe uterine cramping in a 35-year-old female after SCIT to environmental allergens, dosed at 0.1 mL (1:1) of trees, grasses, dust mite, cat, dog, and weeds. She improved with three injections of 0.3 mg epinephrine with complete resolution of symptoms [6]. Another abstract reported uterine cramping with a patient undergoing SCIT at 0.2 mL (1:10) for aeroallergens and fire ant allergies [1]. One article reported uterine cramping after SCIT for hymenoptera venom [7]. Bleeding per vagina was seen alongside uterine cramping in SCIT for *Parietaria* [8] and with pollen, grasses, and dog hair [2]. Interestingly, one article reported lower abdominal cramping with airway reactivity after sublingual immunotherapy for grass pollen [9].

Mechanisms for uterine cramping in anaphylaxis are poorly explained in the literature. The degranulation of basophils and mast cells releases cytokines and other molecules that induce and potentiate allergic reactions [10]. It is hypothesized that the release of these irritative molecules in the myometrium of the uterus could cause a contraction of uterine muscle fibers, resulting in severe menstrual-like cramping [10]. Furthermore, calcium is a known player in physiological muscle contraction, eliciting sarcomere coupling and uncoupling. Thus, coexisting hypocalcemia at the time of uterine cramping may be a contributing factor in the presentation of anaphylaxis [7]. In females with endometriosis, endometrial implants may have a role in the systemic allergic reaction, as implants may contain mast cells [10]. Furthermore, the location of the endometrial implants may be variable, such as the surface of the uterus and abdomen, with potentially enhanced pain sensation if located close to nerve endings [10].

Immediate treatment in the event of anaphylaxis is epinephrine and antihistamines. However, several other treatments have been hypothesized to aid in the event of this medical emergency. Terbutaline, a beta-agonist, was mentioned in multiple reports to terminate uterine cramping [1,10,11]. It is commonly used during labor and delivery to cease uterine contractions if the fetal blood supply is compromised due to compression of fetal vessels during a strong and/or sustained uterine contraction. Furthermore, due to the beta-agonist qualities of this drug, it theoretically could help reduce bronchospasm in the event of severe airway reactivity during the allergic reaction [10,11]. Nonsteroidal anti-inflammatory drugs (NSAIDs) are also commonly used to reduce uterine cramping by blocking prostaglandin production. However, it is cautioned if used during oral immunotherapy, as NSAIDs may increase gut barrier permeability and allow an increased amount of allergen uptake by the intestine [10]. The role of methylene blue has been hypothesized to relax the myometrium in ex vivo experiments; however, we could not find literature using this compound to treat uterine cramping in humans [12]. A possible mechanism for this drug may be in its ability to restore abnormal vasodilation seen in anaphylaxis. There is limited evidence to support the use of oral cromolyn (due to poor gastric absorption), glucagon, nitroglycerin, and corticosteroids for uterine cramping [10]. Lastly, calcium infusion was shown to improve cramping in a patient with known hypocalcemia secondary to total thyroidectomy [7]. In contrast, the literature states that calcium channel blockers (in patients with normal calcium levels) are first-line tocolytics; however, they have never been reported to be used in the context of uterine anaphylaxis [10]. The use of both supplemental calcium and calcium-blocking agents suggests that the relationship between calcium and uterine muscle contraction is a unique and delicate balance that may not yet be fully understood.

## Conclusions

Subcutaneous immunotherapy (SCIT) is commonly known as “allergy shots.” We discuss the details of four patients with uterine cramping, a rare complication of SCIT. To the best of our knowledge, this is the largest case series at this time. We detail the allergens that triggered the reaction, the dosages, and the immediate treatment for hypersensitivity. Our literature search revealed that publications detailing uterine cramping to environmental allergen SCIT are severely limited, with many being abstracts without a full literature review. Thus, our aim for this paper is to report our findings with a deep literature review of the existing reports of this phenomenon, along with potential mechanisms and treatments that could provide insight for clinicians and patients experiencing this condition. To the best of our knowledge, this is the largest case series reporting this symptom after SCIT administration.
